# Risk Factors for Early Childhood Caries in Toddlers: An Institution-based Study

**DOI:** 10.7759/cureus.7516

**Published:** 2020-04-02

**Authors:** Akila Ganesh, Vandana Sampath, Banu Priya Sivanandam, Sangeetha H, Archana Ramesh

**Affiliations:** 1 Public Health Dentistry, Sri Ramachandra Institute of Higher Education and Research, Chennai, IND; 2 Oral Pathology, Sri Ramachandra Institute of Higher Education and Research, Chennai, IND; 3 Dentistry, Sri Ramachandra Institute of Higher Education and Research, Chennai, IND; 4 Dentistry, Sri Ramachandra Institue of Higher Education and Research, Chennai, IND

**Keywords:** toddlers, sweet score, icdas, ecc

## Abstract

Background

Tooth decay experience among toddlers and preschoolers is of epidemic proportions worldwide and dental caries still remains an important childhood disease affecting a considerable part of this population. Though the prevalence of Early Childhood Caries (ECC) is associated with several risk factors such as feeding and oral hygiene practices, Streptococcus mutans levels, socioeconomic status (SES), etc., it is suggested that these factors should be studied adequately to aid in the early prevention and management of ECC.

Objective

The objectives of the study were to: a) evaluate the distribution of ECC, b) study the role of SES in the occurrence of ECC, c) record the variations in feeding and dietary practices along with oral hygiene practices and d) Correlate the sweet score with ECC.

Materials and Methods

This cross-sectional observational study was conducted over a period of 6 months among 100 toddlers (12-36 months) attending the Pediatric outpatient department of a single medical institution in Chennai, India. The study consisted of an intra-oral examination followed by a face to face interview of the mothers of the children using a validated structured oral health questionnaire.

Results

SES and ECC were negatively correlated with statistically significant association. Majority of the subjects did not follow any oral hygiene practices before teeth erupted; few subjects used tooth brush and tooth paste after teeth erupted and followed oral hygiene practices once a day. Statistically significant positive correlation with ICDAS scores was noted in relation to the sweet score and the frequency of intake of sweet foods, candy, etc. Cavitated lesions were more common than non-cavitated lesions and majority of the posterior teeth had ICDAS score 4.

Conclusion

Healthcare providers for children must be well informed on the etiology and risk factors of ECC and guide children for their first dental visit within one year of age.

## Introduction

Dental caries experience among toddlers and preschoolers is of epidemic proportions worldwide. Dental caries continues to be a major public health concern, affecting 60% to 90% of school-aged children and adults in both industrialized and developing countries [1]. American Academy of Pediatric Dentistry, 2010 defines Early childhood caries (ECC) as ‘the most common chronic disease condition in childhood. It involves the presence of one or more decayed (non cavitated or cavitated lesions), missing (due to caries), or filled tooth surfaces in any primary tooth in a child of age 72 months or younger [2].

The risk factors related to ECC can be biological, behavioral or socioeconomic contributors to the caries process. The most significant factors contributing to the risk of developing the disease include feeding and oral hygiene practices, levels of Streptococcus mutans, various dental problems in parents or caregivers, the socioeconomic status, and the time of the first dental visit [3]. A systematic review by Ganesh et al. reported the average prevalence of ECC in India as 49.6%. In a systematic review, Valaitis et al. stated that breastfeeding for over a year and during night time after tooth eruption may lead to ECC [4,5].

Apart from being prone to developing new carious lesions during adolescence, children with neglected and untreated ECC are also prone to complications such as low self-esteem, altered eating habits, dental pain, and absence from school, which in turn unfavourably affect the children’s oral health-related quality of life and overall well-being [6]. It is suggested that the etiology and associated factors of this major public health problem affecting developing and industrialized countries should be studied adequately to assist in the early prevention and management of ECC.

The objectives of the study were to a) evaluate the distribution of ECC b) study the role of SES in the occurrence of ECC c) record the variations in feeding and dietary practices along with oral hygiene practices d) Correlate the sweet score with ECC.

## Materials and methods

This cross-sectional observational study was conducted over a period of 6 months among toddlers (12-36 months) attending the Pediatric outpatient department of a single medical institution in Chennai, India. Chennai district is the administrative capital of the state of Tamil Nadu and the fourth most populous metropolitan city in India. Children of 0-6 years of age form 9.88% of the district’s population [7]. The sample size was calculated based on a study done by Jose et al. with a relative precision of 5% and 95% confidence interval and was estimated to be 81 [8]. For the purpose of minimising error, it was rounded off to 100.

Before commencement of the study, the mothers of the toddlers were invited to voluntarily participate and the objectives of the study were explained to ensure full cooperation. Following their verbal consent for participation, they were requested to sign an informed consent form. Parents were assured they were free to withdraw at any point in time and that there will be no prejudice against the children who had opted to not participate in the study. Awareness was raised to all the parents regarding the appropriate oral hygiene measures and the recommended dental treatment for their children.

Intra-examiner variability was measured by carrying out a reproducibility test where 20 toddlers were examined twice and results were compared until acceptable consistency was achieved. Toddlers aged 12-36 months with the presence of early childhood caries on oral examination were included in the study while children with serious medical problems, systemic disorders or developmental anomalies and children with mothers who refused to participate were excluded.

On the days designated for examination, toddlers attending the outpatient department and fulfilling the inclusion criteria were included in the study until the desired sample size was reached. The data collection for the study was carried out by AG in 2 stages:

Intra-oral examination

Type III clinical examination (ADA specification) was carried out and the children were visually examined for caries according to WHO criteria, 1997 [9]. The dental caries was recorded as per the advanced International Caries Detection and Assessment System4 (ICDAS II). The ICDAS, developed for use in clinical practice, research and epidemiological surveys is based on visual inspection and used to detect cavitated and non cavitated lesions with accepted reliability [10] .

Oral health questionnaire

The mothers of the toddlers, chosen for the study were interviewed face-to-face using a structured questionnaire. The questionnaire framed in English and translated to the vernacular language was face and content validated by 3 experts in the field of paediatrics and public health. The questionnaire consisted of four domains; socio-demographic and socio-economic factors, feeding practices, dietary habits and oral hygiene measures.

Socio-demographic and Socio-economic factors

Socio-demographic data such as name, gender, age and contact number were recorded. Socio-economic details such as educational level, occupation and family income per month were provided by the parents and their socio-economic status (SES) was calculated using the Kuppuswamy’s scale which is the most widely used and popular scale to assess SES in India [11].

Feeding practices

Feeding practices were recorded using a modified form of the questionnaire used in Infant Feeding Practices study II, conducted by Food and Drug Administration (FDA) and Centers for Disease Control and Prevention (CDC) [12]. This domain included questions about the toddler’s feeding practices and frequency of feeding per day during the past 7 days. History of bedtime feeding practices, age of first formula feed, consumption of juices, sweets, dairy foods, cereals etc. were also recorded.

Dietary Habits

The diet diary was recorded for sucrose consumption according to the method described by Nizel and Papas [13]. Dietary and nutritional information involved questions regarding the form and frequency of food consumption which were categorized as liquid/ solid and sticky/ slowly dissolving. The 24-hour diet recall was documented and the sweet score was calculated from the obtained diet chart.

Oral hygiene practices

The structured questionnaire contained the most frequent and significant risk factors for ECC. The mothers were asked about the toddler’s method and frequency of cleaning the oral cavity. The oral hygiene methods practiced both before and after tooth eruption were recorded. The statistical analysis was done using SPSS 19 (IBM). For continuous variables, the descriptive statistics were expressed in terms of mean and standard deviation and for categorical variables, it was expressed in terms of frequency and percentage. Inferential statistics was done based on the normality of the data. Kruskal Wallis Test and Mann Whitney U test were used to compare the differences between the independent variables. Correlation was assessed using Spearman’s Rank Order Correlation Coefficient test. p < 0.05 was considered to be statistically significant.

## Results

The study was carried out among 100 toddlers between the age of 12-36 months from Chennai, India. The mean age of the selected subjects was 29 months. Equal distribution of boys and girls was observed in the study.

Socio-economic status

When assessing the socio economic status, 43% of the parents belonged to the lower middle class and 35% belonged to the upper middle class. The SES was negatively correlated with the number of carious teeth; and the income factor showed statistically significant negative correlation (Table 1).

**Table 1:** Correlation between various risk factors with carious teeth based on ICDAS scores

	Education N=100	Occupation N=100	Income N=100	SES N=100	Sweet score N=100	100% fruit/ vegetable juice N=100	Baby cereal N=100	Fruit N=100	Vegetables N=100	Sweet foods N=100
n	100	100	100	100	100	96	96	94	83	42
Spearman’s Rank Correlation Coefficient	0.03	-0.05	-0.22	-0.05	0.32	-0.02	0.05	0.09	-0.05	0.31
p value	0.75	0.62	0.03	0.62	0.001	0.82	0.61	0.39	0.65	0.04

Feeding Practices

**Figure 1:**
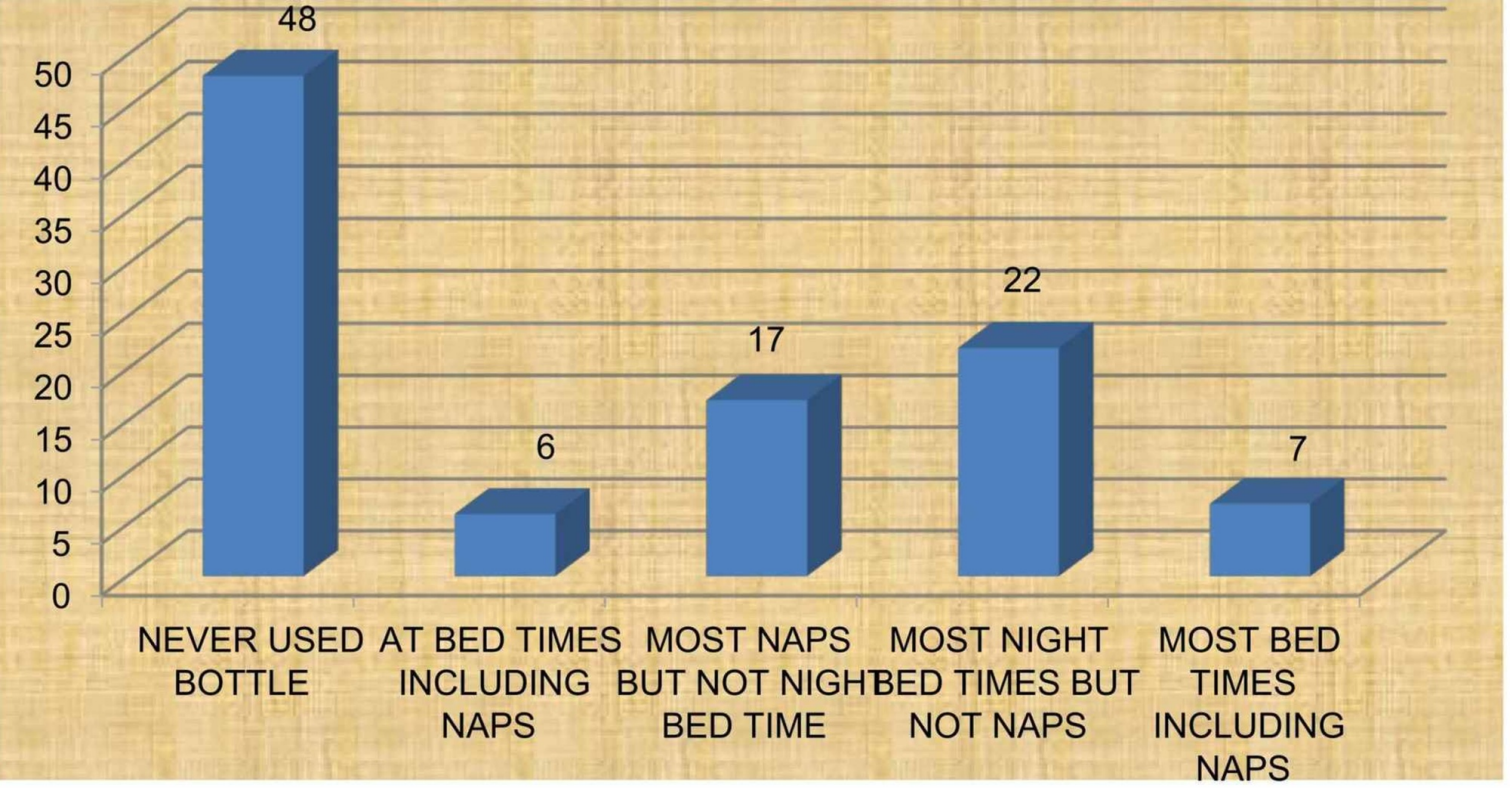
Assessment of feeding practices

Breastfeeding was reported in 94% of the subjects on an average of 5.63 feedings per day. Of the 52% who reported bottle feeding, 22% were fed at night bedtimes, but not during nap-times (Figure 1). History of bottle feeding was statistically significant with dental caries (p=0.04). Formula and cow’s milk were consumed 2-3 times per day. Formula milk was first fed at a mean age of 11.6 months and 45% of the subjects consumed milk with added sweeteners.

Oral Hygiene Practices

**Figure 2:**
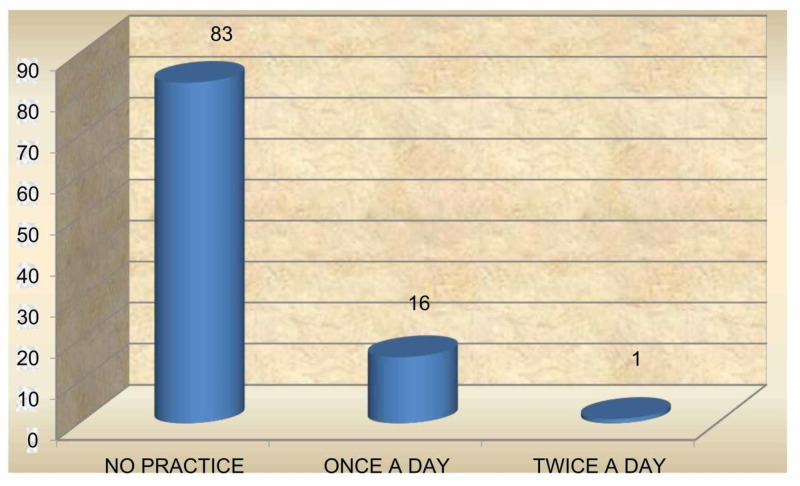
Frequency of oral hygiene practices before teeth eruption

**Figure 3:**
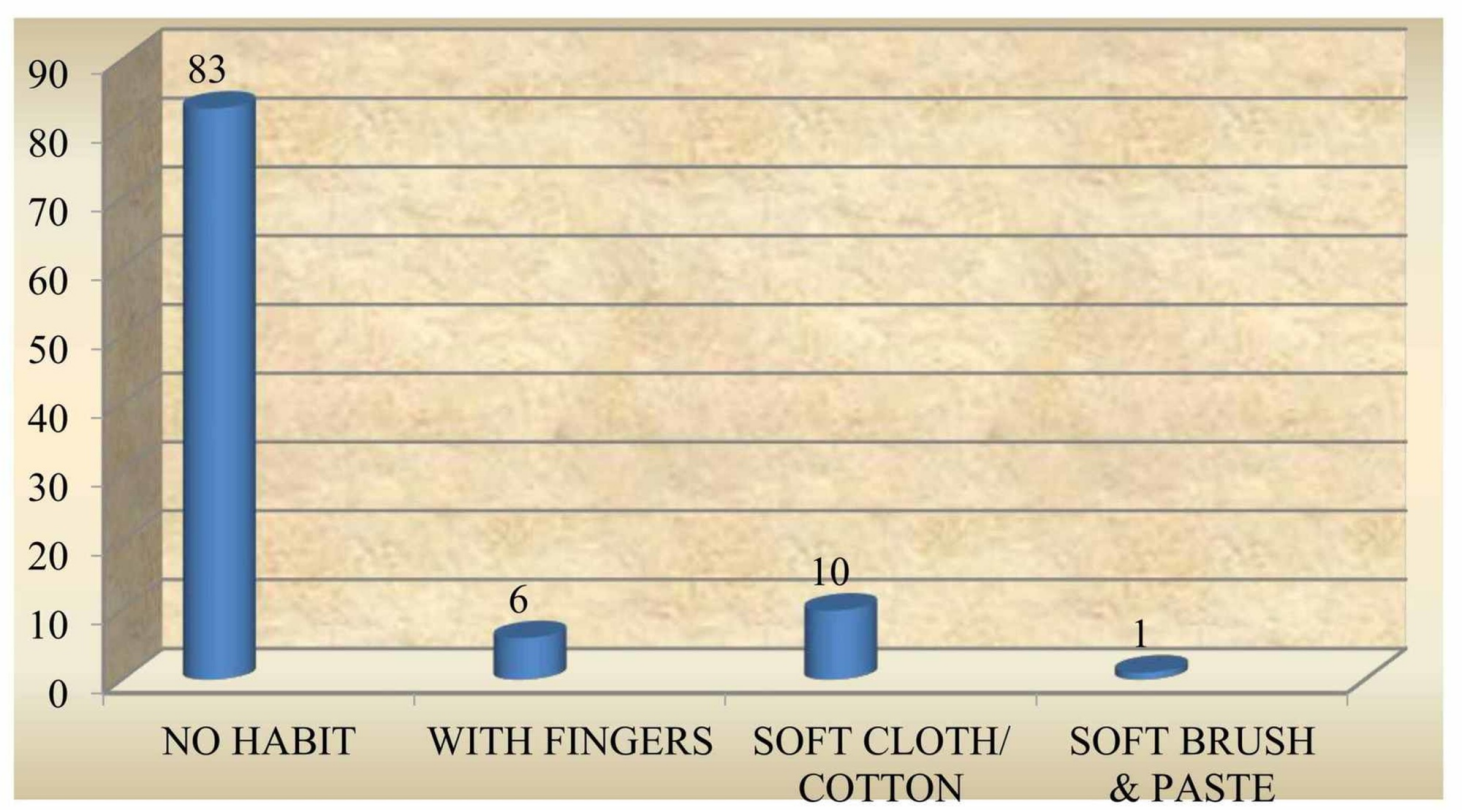
Oral hygiene practices before teeth eruption

**Figure 4:**
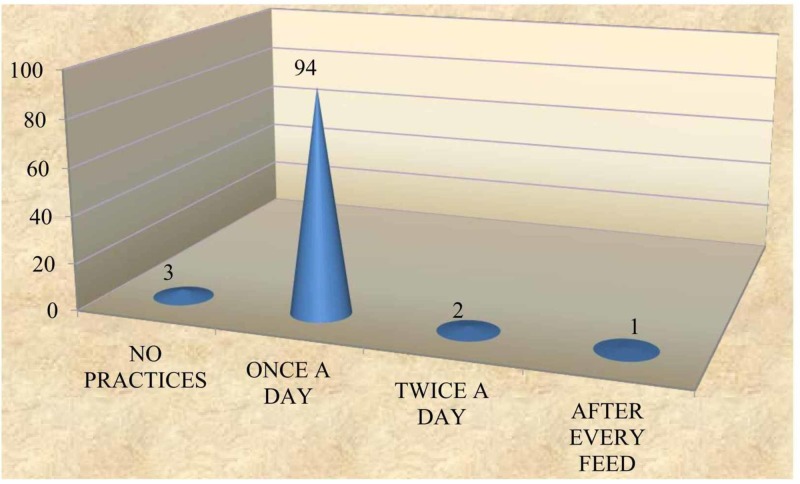
Frequency of oral hygiene practices after teeth eruption

**Figure 5:**
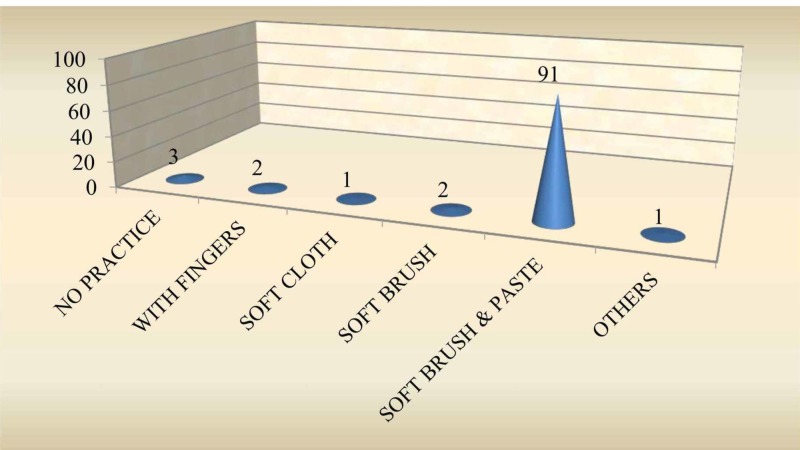
Oral hygiene practices after teeth eruption

A majority (83%) of the subjects did not practice oral hygiene measures before teeth erupted, while 16% cleaned once a day and only 1% cleaned twice a day (Figure 2). When assessing the method of maintaining oral hygiene, it was found that of the 17%, who cleaned their gum pads, 10% used a soft cloth /cotton, 6% used fingers and 1% used soft brush and paste (Figure 3). Furthermore, 94% of subjects had a habit of cleaning the teeth once a day with a soft brush and tooth paste after teeth eruption; 2% of subjects cleaned the teeth twice a day and 1% cleaned the teeth after a feed. However, 3% reported lack of oral hygiene practices even after tooth eruption (Figure 4); 91% of the subjects used a soft brush and toothpaste to clean the teeth and gum pads (Figure 5).

Carious lesion and dietary practices

**Figure 6:**
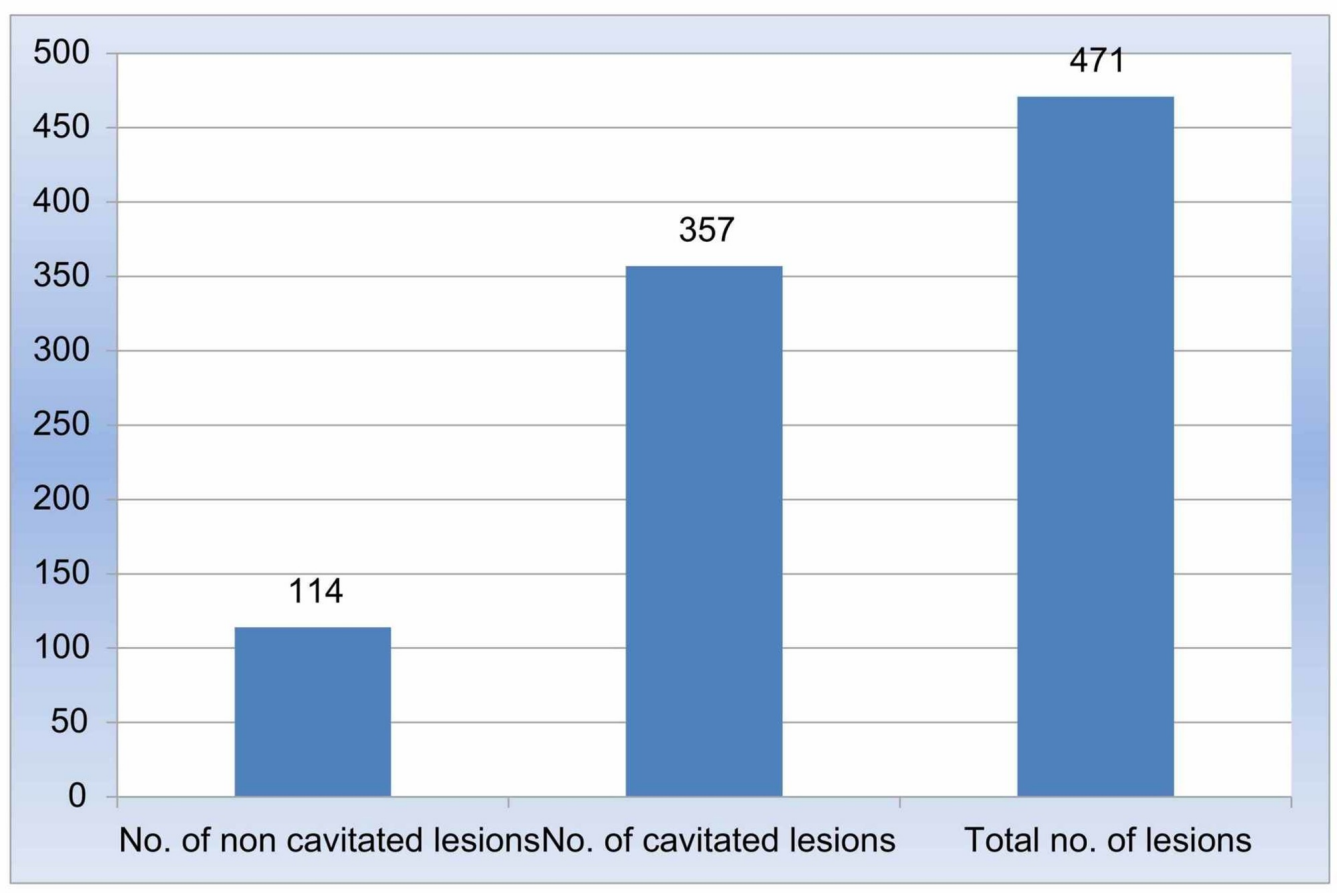
Cavitated versus non-cavitated lesions

**Figure 7:**
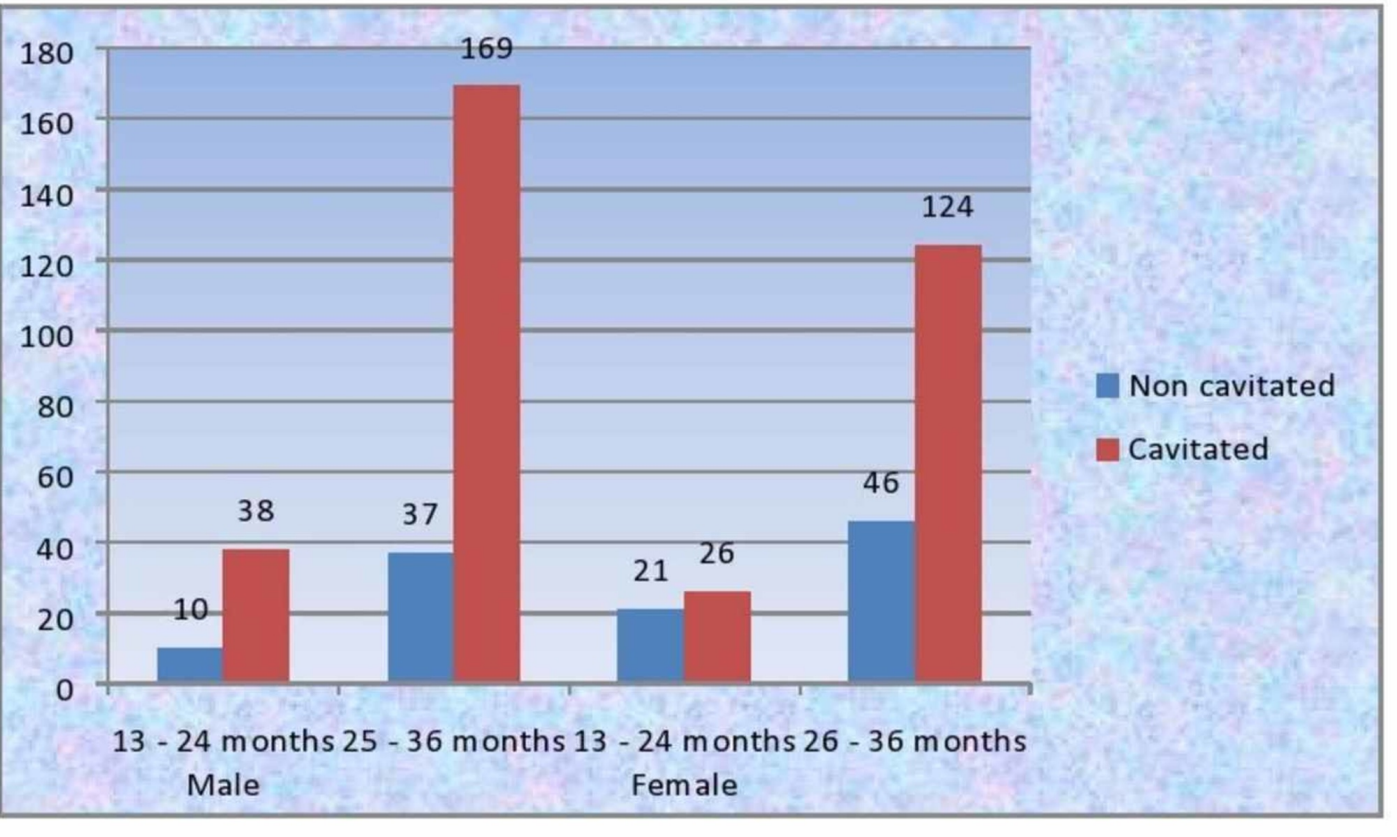
Distribution of carious lesions

While assessing the ICDAS scores, majority of the central incisors had ICDAS score 4, lateral incisors had ICDAS score 6, followed by canines with a score of 2 and the molars with a score of 5. Of the 471 lesions reported, 357 were cavitated and 114 were non cavitated (Figure 6). A positive correlation was reported between the age and carious lesions among boys and girls. Gender comparison revealed that boys showed a higher percentage of cavitated lesions, while girls showed a higher tendency for non cavitated lesions (Figure 7). The most commonly affected teeth surfaces were the labial and the occlusal surfaces of the anteriors and posteriors respectively. Intake of sweet foods, candies, cookies, etc. showed a positive correlation with the number of carious lesions. The sweet score was also positively correlated with the ICDAS scores. The above results were statistically significant (Table 1).

## Discussion

ECC is a cause of social and economic burden globally and is believed to affect the child’s quality of life. Hence it is important for health care workers to extend and channelize their efforts towards the prevention and management of this disease. For this reason, a deeper understanding of the risk factors involved in ECC is of paramount importance. A systematic review by Harris et al. described almost 90 risk factors for ECC [14]. Kirthiga et al. concluded the strongest and significant secondary risk factors associated with early childhood caries in high- or upper-middle-income categories to be the presence of enamel defects, high levels of mutans streptococci, presence of dental caries, frequent consumption of sweetened foods, poor oral hygiene, and the presence of visible plaque [15]. According to available evidence, the risk factors for caries varies among children with different backgrounds, and are also affected by the study designs, participants, and statistical analysis techniques used [16]. In the present study, the most commonly reported and agreed upon factors in literature such as SES, dietary and feeding habits and oral hygiene practices were chosen [3-6].

The age range of 12-36 months was chosen to expose the primary dentition to the oral environment for a minimum period of 6 months post-eruption. In the present study, the gender distribution of ECC among boys and girls was found to be equal which was in contrast to studies conducted by Maciel et al. and Kabil et al. who reported a higher prevalence in boys compared to girls, with highly significant results (p < 0.001) [17-18].

The inverse relation between SES and ECC reported in the current study was similar to other studies by Tyagi et al. , Plutzer et al. and Kabil et al. that ascribed the unawareness regarding oral health care for children of parents with low socio-economic groups to this finding [18-20]. Delay in dental visits and inconsideration of oral health as a priority could be other possible reasons.

Shrutha et al. reported breast feeding for 5-10 times in 60% of the children and stated that caries prevalence was statistically significant (p<0.05) among those who were breast fed for longer duration, during night time, those falling asleep with bottle and those fed with additional sugar in milk [21]. Tyagi et al. and Mohebbi et al. also observed increased prevalence of ECC in children who had a habit of taking a feeding bottle to bed at night [19, 22]. Similar results were reported in the present study. This finding could possibly be due to the fact that decreased salivary flow during sleep time reduces the liquid carbohydrates clearance from the oral cavity, acting as a determinant in caries initiation. However, Reisine et al. stated the practice of bottle feeding, its duration in terms of age of child, bottle feeding during night and contents of the bottle did not show any significant association with ECC [23]. These results were similar to findings of Dini et al. [24]. Furthermore, Tiberia et al., Perera et al. and Kabil et al. reported no remarkable difference between children who were bottle fed or breast fed in terms of caries experience [19, 25-26]. These studies substantiate the findings that the mode of feeding in the disease process of ECC plays a less significant role than the interaction of intraoral bacterial load and risk factors. Hence, the role of bottle feeding in the occurrence of ECC is controversial and more research is needed to justify it.

The positive association of sugar consumption and caries experience observed in the current study was consistent with most of the available literature including the systematic review done by Harris et al. [14, 27-30]. He reported the high sucrose content in solid or sticky foods and sweetened beverages to be responsible for caries development.

The novel system of ICDAS II, used in the study is an accurate and improved system with good reproducibility to detect incipient caries as well as to understand the severity and activity state of the lesions [10]. This system paves the road for further research to compare different studies globally with increased accuracy .The data collection of these factors was executed by direct face to face interviewing of the mothers rather than using a self administered form in order to avoid misinterpretation of the questions. Despite these merits, since the present study was cross-sectional with a limited sample size, causal relationships could not be established and the associations observed may be due to other unexplored confounding factors.

## Conclusions

SES was inversely related to dental caries. History of bottle feeding and frequency of sweet intake was directly related to caries experience. Cavitated lesions were more prevalent in males with labial and occlusal surfaces being most commonly affected. Despite the numerous risk factors reported for ECC, this condition can be prevented if appropriate measures are applied. The first dental examination between six months and one year of age is often impractical, due to the lack of awareness among the general population. Hence, there is a great need for preventive efforts by the child’s healthcare providers (e.g. pediatricians, family physicians, nurses etc) to be well informed on the etiology and risk factors of ECC, and thus play a crucial role in guiding the children for their first dental visit within one year of age.
